# The effect of multisensory semantic congruency on unisensory object recognition in schizophrenia

**DOI:** 10.3389/fpsyt.2023.1246879

**Published:** 2023-11-01

**Authors:** Erfan Ghaneirad, Anna Borgolte, Christopher Sinke, Anja Čuš, Stefan Bleich, Gregor R. Szycik

**Affiliations:** ^1^Department of Psychiatry, Social Psychiatry and Psychotherapy, Hannover Medical School, Hanover, Germany; ^2^Department of Psychiatry, Social Psychiatry and Psychotherapy, Division of Clinical Psychology and Sexual Medicine, Hannover Medical School, Hannover, Germany; ^3^Center for Systems Neuroscience, University of Veterinary Medicine, Hanover, Germany

**Keywords:** schizophrenia, multisensory memory, multisensory integration, multisensory object recognition, congruency effect

## Abstract

Multisensory, as opposed to unisensory processing of stimuli, has been found to enhance the performance (e.g., reaction time, accuracy, and discrimination) of healthy individuals across various tasks. However, this enhancement is not as pronounced in patients with schizophrenia (SZ), indicating impaired multisensory integration (MSI) in these individuals. To the best of our knowledge, no study has yet investigated the impact of MSI deficits in the context of working memory, a domain highly reliant on multisensory processing and substantially impaired in schizophrenia. To address this research gap, we employed two adopted versions of the continuous object recognition task to investigate the effect of single-trail multisensory encoding on subsequent object recognition in 21 schizophrenia patients and 21 healthy controls (HC). Participants were tasked with discriminating between initial and repeated presentations. For the initial presentations, half of the stimuli were audiovisual pairings, while the other half were presented unimodal. The task-relevant stimuli were then presented a second time in a unisensory manner (either auditory stimuli in the auditory task or visual stimuli in the visual task). To explore the impact of semantic context on multisensory encoding, half of the audiovisual pairings were selected to be semantically congruent, while the remaining pairs were not semantically related to each other. Consistent with prior studies, our findings demonstrated that the impact of single-trial multisensory presentation during encoding remains discernible during subsequent object recognition. This influence could be distinguished based on the semantic congruity between the auditory and visual stimuli presented during the encoding. This effect was more robust in the auditory task. In the auditory task, when congruent multisensory pairings were encoded, both participant groups demonstrated a multisensory facilitation effect. This effect resulted in improved accuracy and RT performance. Regarding incongruent audiovisual encoding, as expected, HC did not demonstrate an evident multisensory facilitation effect on memory performance. In contrast, SZs exhibited an atypically accelerated reaction time during the subsequent auditory object recognition. Based on the predictive coding model we propose that this observed deviations indicate a reduced semantic modulatory effect and anomalous predictive errors signaling, particularly in the context of conflicting cross-modal sensory inputs in SZ.

## 1. Introduction

Cognitive dysfunctions is one of the most significant impairments in patients with schizophrenia (SZ) negatively affecting their occupational, social and economic functioning (1). Memory disruptions are a prominent among the findings of impaired higher-level cognitive processing in SZ (2, 3). Accumulating evidence suggests that the impairment is profound and affects most subtypes of memory (4–10). Patients show impairments especially in their ability to encode contextual information, including information associated with target memory as well as retrieving target information using contextual information (4, 11–14).

Experimental paradigms designed to study memory functions traditionally used unimodal stimuli presentation for encoding (e.g., present auditory or visual objects). However, in everyday life we experience and encode our environment through simultaneous inputs from multiple sensory organs. An effective integration of sensory modalities is substantial for generating a coherent and meaningful perception and improves perceptual acuity (15, 16), detection (17, 18), recognition (19, 20) and response speed (21, 22). Accordingly, influential cognitive models of memory (23–28) have argued that the ability to integrate features during encoding enhances the memory performance. Feature integration mostly does not occurs within a single sense; instead, it requires combining inputs from multiple senses (e.g., visual and auditory features) to form a coherent and meaningful perceptual object.

Multisensory integration (MSI) has increasingly been found to rely upon neural communication both within specific cortical modules and across broad neural networks (29–31). Similarly, the recent pathophysiological theories of schizophrenia highlight the role of disrupted neural communication and abnormalities in the connection between neurons and neuronal populations (32, 33). Additionally, the idea of MSI abnormalities in SZ found support from recent experimental data obtained from various paradigms (34–38). For instance, in a phenomenon referred to as sound-induced double flash illusion (39), where a rapid presentation of a single visual stimulus (flash), synchronized with two auditory stimuli (beeps) leads to the deceptive perception of two visual stimuli among healthy participants. However, patients with schizophrenia reported less illusory visual perceptions compared to healthy controls (36). This result can be elucidated by the reduced impact of auditory input on visual perception in these patients. Furthermore, Williams and colleagues (34) found impairments in intersensory facilitation, i.e., longer reaction times for the detection of simple, temporally congruent audio-visual targets compared to unisensory targets and a positive relationship between these impairments and psychotic symptoms. In addition, compared to healthy subjects, patients with SZ showed a reduced sensitivity to asynchrony of multimodal stimuli (40, 41) and require a more extended temporal interval to accurately detect asynchronous stimuli (42). Evidence about MSI impairments in SZ is not limited to simple stimuli; it has also been reported in studies using more complex and socially relevant stimuli like audiovisual speech (43, 44).

The previous studies indicating abnormalities in MSI in SZ predominantly investigated multisensory processes at the early stages of perception. However, it remains unclear how multisensory deficits can cascade into higher-order cognitive abnormalities that characterize this disorder (45). Previous research employing animal models and early developmental studies suggests that multisensory processing plays a fundamental role in the acquisition of advanced cognitive functions (46, 47). Multisensory maturation has been linked with numerous cognitive and perceptual abilities, from memory and attention to numerical discrimination and abstract rule learning (48–56). A logical extension of this scaffolding theory posits that disruptions in sensory functioning are likely to have far-reaching effects across different cognitive domains (57). Conversely, recent research suggests that higher-order cognitive processes can also influence the processing of sensory information (58). This phenomenon, referred to as the “top-down effect,” demonstrate that our anticipations or internal models possess the capacity to influence multisensory perception, for instance, by directing attention towards task-relevant stimuli (58). A better understanding of how low-level perceptual processes and higher-order processes are interconnected would provide a more comprehensive understanding of the characteristics and nature of both systems.

Considering the co-existing MSI deficits and memory dysfunctions in SZ on the one side and evidence about their interrelatedness (59, 60) on the other side, the current study investigates the effect of deviations in audiovisual integration during encoding on subsequent unisensory object recognition tasks in this group of patients. To achieve this, two adapted versions of the continuous recognition task (61) were utilized. Both versions of the task shared a same structure, differing solely in the modality that participants attended to (auditory or visual). Participants were engaged in a task that involved distinguishing between initial (new) and repeated (old) presentations of stimuli, which were intermixed within a continuous recognition task. In this context, half of the initial presentations were multisensory, while the subsequent repetitions exclusively comprised task-relevant unisensory stimuli. The rationale behind choosing this task was to enable an exploration of the top-down influence exerted by memory-based semantic associations during the encoding process. This exploration was facilitated through the manipulation of multisensory presentations with semantic variations, employing naturalistic real-world objects to elicit long-term, semantic associations between sensory inputs (19). Hence, half of the multisensory presentations were selected to be congruent (e.g., a drawing of a pig with the sound of grunting), whereas the remaining half were chosen to be incongruent (e.g., combining a drawing of a church with the sound of a ringing phone).

Previous studies using continuous recognition tasks with healthy subjects have demonstrated that the memory of objects, encoded in the audiovisual context can be more robust than that of objects encoded exclusively in a visual or an auditory context (61–64). Lehman and Murray (20) found improved object discrimination accuracy in multisensory encoding conditions compared to unimodal encoding. Specifically, Initial presentation of semantically congruent pairing has been shown to improve subsequent retrieval, whereas being initially presented with incongruent audiovisual pairing negatively impacted memory performance (19).

We hypothesize that patients with SZ will exhibit a distinctive performance pattern due to the deficits in MSI during the encoding process. Specifically, it is anticipated that patients will demonstrate a decrease in multisensory facilitation effect in congruent condition, as well as a diminished negative influence of incongruent encoding on their performance, as measured by accuracy rate (ACC) and reaction time (RT) in subsequent unisensory object recognition when compared to control subjects.

## 2. Methods

### 2.1. Participants

This study included 21 adult patients (8 female) who fulfilled the DSM-5 criteria (65) for schizophrenia spectrum disorders [schizophrenia (*n* = 18), delusional disorder (*n* = 1), schizoaffective disorder (*n* = 2)]. Patients were recruited from both the in-patient and out-patient services of the department of psychiatry at Hannover Medical School. Following consulting with the treating psychiatrist, patients with acute and severe psychotic symptoms and/or unstable medication were not contacted to be invited for participation in the study. Additionally, a total of 21 (14 female) healthy adult controls (HC) were recruited via local community advertisements, with groups being matched for age, gender and estimated verbal IQ as assessed by the MWT-B (66). All participants had normal or corrected-to-normal vision and reported normal hearing. Furthermore, (66) all participants were native speakers of German (see Table 1 for detailed sociodemographic characteristics of the sample) and provided informed consent before participation. Both the patient group and the control group were screened with the German version of the Structured Clinical Interview for DSM-5 Clinician Version (67) and the Structured Clinical Interview for DSM-5 Personality Disorder (68). In order to measure positive and negative symptoms of psychotic disorders, the patients were also interviewed with the positive and negative syndrome scale (PANSS) for schizophrenia (69). All patients received atypical antipsychotic medication. The patient’s diagnosis, PANSS scores and medication are shown in Table 2. All participants in the control group verbally reported that they had not experienced any diagnosed psychiatric disorders in the past. The general exclusion criteria were diagnosed neurological disorders, as well as active drug or alcohol within 3 months preceding the assessment. After the diagnostic session, subjects who fulfilled the inclusion criteria were invited to participate in two experimental sessions separated by at least 7 days. In each session, they completed either the auditory or the visual task in a counterbalanced order.

**Table 1:** Sociodemographic characteristics of sample.

	SZ		HC		Analysis		
	*N*/*M*	SD	*N*/*M*	SD	*t* or *χ*^2^	*df*	*p*
Gender					3.44	1	0.064
Female	8		14				
Male	13		7				
Age (years)	37.86	10.30	31.90	9.98	−0.190	40	0.064
Educational level*						40	0.381
Low	13		6				
Medium	5		12				
High	3		3				
MWT-B	100.86	11.46	104.52	11.43	1.038	40	0.305

*Low level: secondary level 1 (5–9 school years); Medium level: secondary level 2 (11–12 school years); High level: higher education.

**Table 2:** Patient’s diagnosis, PANSS scores and medication.

	*N*	%	M	*SD*
**Diagnosis**				
Paranoid schizophrenia	18	86.0		
Delusional disorders	1	4.5		
Schizoaffective disorders	2	9.5		
**PANSS subscale**				
Negative symptoms			14.81	5.46
Positive symptoms			15.43	6.00
General symptoms			32.48	6.40
Total score			62.71	14.04
Chlorpromazine equivalent (mg)			335	246.87

The ethics committee of the Hannover Medical School approved the study. All participants gave written informed consent and received a small monetary compensation for their participation.

### 2.2. Experimental paradigm and stimuli

**Figure 1:**
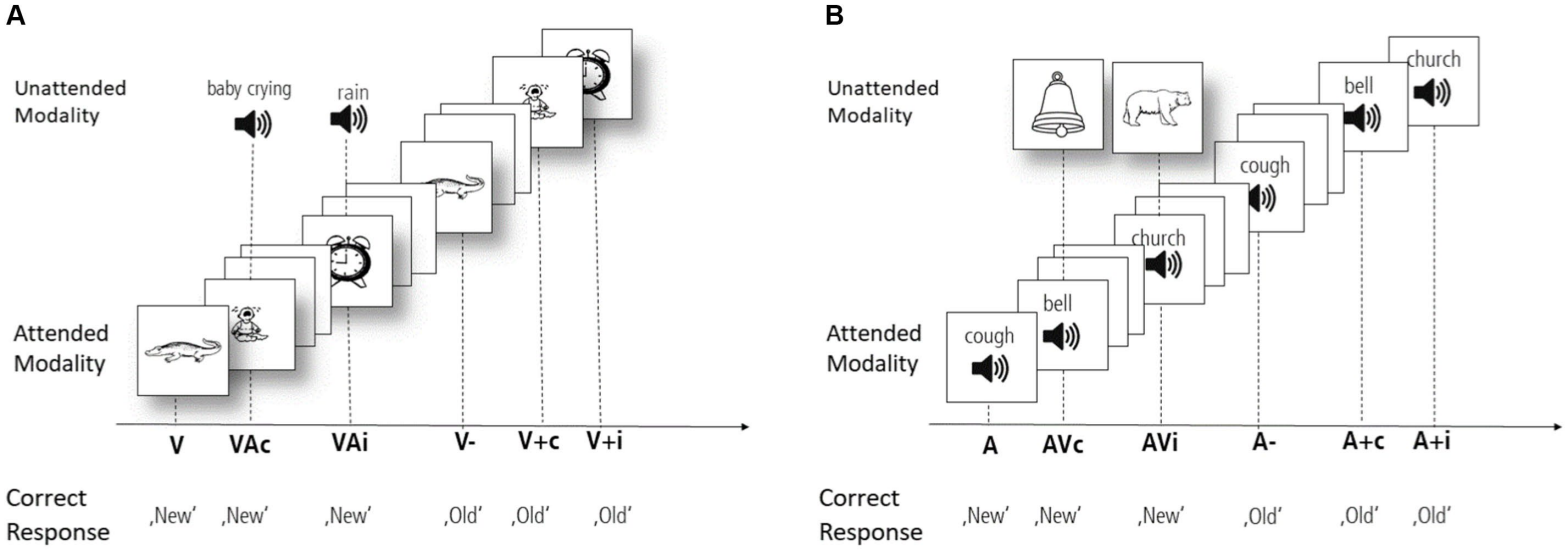
**(A)** Schematic representation of the visual task. The visual task consisted of 288 trials (50% initial). The half of initial presentations were purely visual stimuli (V) and the other half presented audiovisual pairings. Among the multimodal condition, 50% of presentations were semantically congruent (VAc) and 50% were incongruent (VAi). All repeated presentations were unimodal. Upon repetition half of the stimuli (72) were identical to the initial presentation (V−), the other half (72) were unimodal presentations of previously audiovisual pairings; 36 initially congruent presentation (V + c) and 36 initially incongruent presentation (V + i), All stimuli were presented for a duration of 500 ms, followed by a randomized inter-trial interval ranging from 900 to 1.500 ms, during which a fixation cross was displayed on the monitor. Due to space limitations, the inter-trial intervals are not depicted. **(B)** Schematic representation of the auditory task. In the auditory task, the structure remains unchanged, with auditory stimuli as the attended modality and visual stimuli as the unattended modality.

An auditory and a visual version of the continuous recognition task (61) were used in the current study. Participants were instructed to indicate, using their index finger on both hands to press two buttons on a computer keyboard, as quickly and accurately as possible whether an item was presented for the first (new) or second (old) time during each task, while attending to either auditory stimuli (auditory task) or visual stimuli (visual task). Each task involved a total of 288 trials, consisting of 144 initial presentations and 144 repeated presentations. Half of the initial presentations were unisensory stimuli, while the other half comprised audiovisual pairings. Within the audiovisual pairings, 50% were semantically congruent, while the remaining 50% were incongruent. Notably, all repeated presentations were unimodal. To ensure that subjects understood the task’s instruction, a rehearsal block with 10 trials was performed prior to the task. The experiment was conducted in a sound-attenuated chamber. Figure 1 provides a visual representation of the tasks.

#### 2.2.1. Auditory task

Participants were instructed to engage in a forced-choice task, indicating whether the current sound was presented for the first time (new) or the second time (old). They were informed that some sounds are accompanied by a picture, but the old-new decision should be based exclusively on the heard sound. Half of the initial presentations (72 trials) exclusively involved auditory stimuli (A), while the other half (72 trials) consisted of sound-picture pairings (AV). In the AV-condition, half of the sounds were paired with a congruent picture (AVc, resulting in 36 stimuli), while the other half were presented with an incongruent picture (AVi, remaining 36 stimuli). All repeated stimuli were presented solely in the auditory modality. For clarity in this paper, we refer to repetitions of condition A as ‘A-’ and repetitions of the condition AVc as ‘A + c’ and repetitions of AVi as ‘A + i’. In this way, ‘c’ designates congruence between the auditory and visual modalities, while ‘i’ designates incongruent sound-picture pairings.

#### 2.2.2. Visual task

The visual task mirrored the structure of the auditory task. However, unlike the auditory task, participants were instructed to focus on pictures and to determine whether they saw the picture for the first or second time. Correspondingly, half of the initial presentations were only visual (V) while the other half consisted of audiovisual (VA) pairings (congruent or incongruent). All following repetitions were pictures initially presented unimodal (V−), congruent multimodal (V + c) or incongruent multimodal (V + i).

#### 2.2.3. Stimuli

The visual stimuli were black line drawings on a white background presenting a mix of living (e.g., human, animal) and non-living (e.g., church, music instrument) objects. The images were presented centrally on a 21″ computer Monitor [Sony Trinitron Multiscan G520, Sony Electronics Inc., San Diego, CA, United States, with 1,024 × 768-pixel resolution]. All pictures had the same dimensions (585*585 pixel, covering 11° vertically and 11° horizontally of the visual angle). The auditory stimuli were sounds (16-bit stereo, 44,100-Hz digitization) of common objects (e.g., cough, animal, music instrument). The auditory stimuli were presented in a mono mode through two speakers positioned on the left and right side of participants and the volume was adjusted to a comfortable level for each subject.

All stimuli were presented for a duration of 500 ms, followed by a randomized inter-trial interval ranging from 900 to 1.500 ms, during which a fixation cross was displayed on the monitor. In multisensory conditions, visual and auditory stimuli were presented synchronously. To maintain an equitable distribution of old and new stimuli within each task, we controlled the mean number of trials between initial and repeated presentations to be 9 ± 4 stimuli. This strategy was employed to mitigate response-decision bias and to uphold a consistent probability of encountering new and old trials across the entirety of the tasks (61, 62). Incongruent sound-picture pairings were chosen randomly and were reviewed after randomization to ensure that there is no semantical relation between the visual and auditory stimulus in each pairing. The congruent pairings consisted of picture and sound of the same object (e.g., picture of a cat and meowing sound).

The tasks were presented using the E-Prime 2.0 software (Psychology Software Tools, Inc., Pittsburgh, PA, United States). The stimuli were used and validated in previous works (61, 62) and were kindly supplied by Micah Murray and Antonia Thelen.

### 2.3. Data analysis

The behavioral data were analyzed by calculating the mean RT in milliseconds and the ACC (percentage of correct responses) for each subject and condition separately. The accuracy rate was evaluated within a reaction time window between 150 and 1.500 ms after stimulus onset. Only RT of correct responses was considered in the analysis. The row data of RT and ACC are shown in Table 3. The multisensory gain/cost indices for ACC and RT for each task was calculated for repetition trials. It was defined as the accuracy/reaction time difference between repeated presentations of prior multisensory stimuli and repeated unisensory presentations (e.g., formulas 1 and 2 for ACC and RT of multisensory congruent condition in the auditory task). By using these indices, we were able to compare the impact of multisensory memory traces on subsequent unisensory object discrimination beyond the differences caused by general task-related performance differences (visual vs. auditory).


$$\begin{array}{l} \mathrm{Gain}/\mathrm{cost index}\;ACC\%=\%\mathrm{ACCmultisensory}\;Ac\\ \;-\%ACC\;\mathrm{unisensory}(1) \end{array}$$

$$\begin{array}{l} \mathrm{Gain}/\mathrm{cost index}\;RT\;\mathrm{ms}=\mathrm{msRT multisensory}\;Ac\\ \;-\mathrm{msRT unisensory}(2) \end{array}$$

**Table 3:** Raw performance data of accuracy and RT in both tasks for SZ and HC.

	SZ		HC	
	Auditory	Visual	Auditory	Visual
Accuracy %	M (SD)	M (SD)	M (SD)	M (SD)
Unisensory	65.12 (12.9)	82.90 (12.8)	78.4 (9.8)	95.2 (6.6)
Congruent	79.37 (11.4)	80.09 (15.5)	89 (6.4)	95.5 (4.1)
Incongruent	60.81 (15.9)	84.71 (10.85)	74.35(11.87)	94.45 (4.43)
**RT ms**				
Unisensory	1,243 (233.7)	854 (186.6)	1,176 (186.1)	760 (117.7)
Congruent	1,206 (251.5)	857 (188)	1,120 (199.1)	759(125.7)
Incongruent	1,196 (202.9)	850 (182.9)	1,188 (181.8)	754(115.2)

### 2.4. Statistical analysis

Data were analyzed using IBM SPSS Statistics for Windows, version 28 (IBM Corporation, Armonk, NY, United States). We examined the subjects’ performance for possible response bias and excluded participants with an accuracy rate of lower than 50% which led to the exclusion of 5 participants from SZ group and 1 person from HC group in the auditory task as well as 1 healthy participant from the visual task. Subjects who were excluded from the auditory task were considered in the calculations of the visual task if their performance was above 50%, and vice versa. Data was examined for normality of distribution using the Shapiro–Wilk test. The multisensory gain/cost indices for ACC and RT were averaged separately across tasks for each condition and each participant and submitted to a (2 × 2 × 2) mixed analysis of variance (ANOVA) with modality (auditory vs. visual task) and congruency (congruent vs. incongruent) as within-subject-factors and group (SZ vs. HC) as between-subject-factor. Post-hoc tests were adjusted using the Bonferroni correction. Additionally, and in order to ensure significant deviations from zero for gain/cost indices, each index was subjected to a one-tailed independent t-test against a zero matrix. Further, the relationship between the PANSS (positive, negative subscales and the total score) as well as antipsychotic medication with task performance of patients was studied by use of 2-tailed multiple Pearson correlation analysis. To account for multiple comparisons, the Bonferroni correction was applied. Upon confirming the normal distribution assumption for age, a comparison between the two groups was conducted using an independent-sample *t*-test. Additionally, the groups were compared in terms of educational level and gender using Chi-square statistic.

## 3. Results

Both groups did not differ in age, education, gender as well as in IQ as measured through MWT-B. Sociodemographic characteristics of both groups as well as diagnoses, PANSS scores and medication of the patient group are summarized in Tables 1, 2.

**Figure 2:**
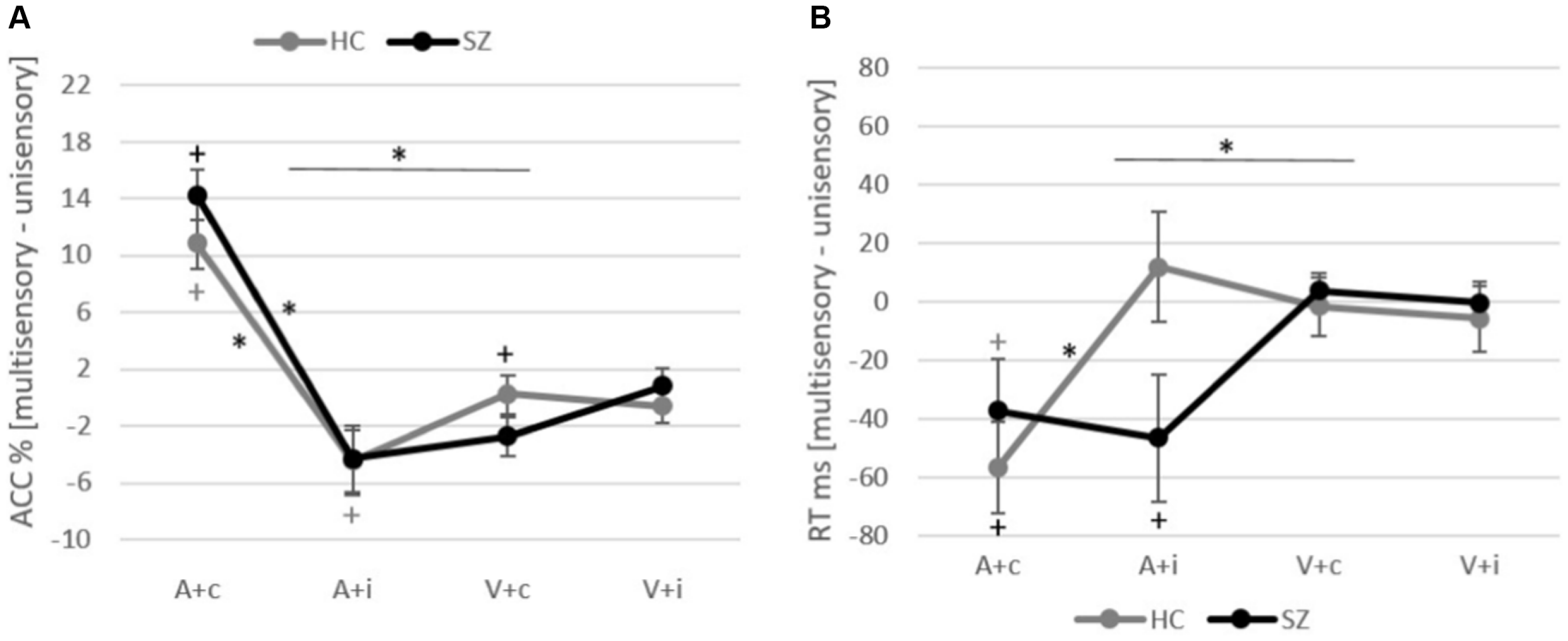
**(A)** Accuracy gain/cost indices and standard error for SZ and HC for both tasks. **(B)** Reaction time gain/cost indices and standard error for SZ and HC for both tasks. Significant effects are marked either with an asterisk for between conditions or with a plus compared to a zero-matrix.

The normality of the data distribution for the gain/cost indices of ACC was assessed using the Shapiro–Wilk test. Results revealed that both A + c (SZ: W = 0.92, *p* = 0.39; HC: W = 0.95, p = 0.39) and A + i (SZ: W = 0.96, *p* = 0.63; HC: W = 0.95, *p* = 0.44) are normally distributed. However, for the V + c condition, the null hypothesis of normality was rejected for both the SZ group (W = 0.87, *p* = 0.03) and the HC group (W = 0.85, *p* = 0.007). Moreover, in the V + i condition, the null hypothesis of normality was rejected for the control group (W = 0.81, *p* = 0.002). The assessments of homogeneity of covariance via Box’s test yielded a non-significant result (*p* = 0.112). Furthermore, homogeneity of variances across all conditions was established by Levene’s test of equality of error variance, with all *p* > 0.05. Considering the robustness of the ANOVA to violations of normality (70–72) a 2 × 2 × 2 mixed ANOVA was performed, with modality (auditory vs. visual) and semantic (congruent vs. incongruent) as within-subject factors, and group (SZ vs. HC) as a between-subject factor on the gain/cost scores. This analysis revealed a significant main effect of modality (*F* (1, 33) = 10.99, *p* = 0.002, *η*_p_^2^ = 0.25), indicating a more robust impact of visual task-irrelevant stimuli (M = 4.08, SD = 6.28) on later object recognition compared to auditory task-irrelevant stimuli (M = −0.563, SD = 4.48). Moreover, a main effect of semantic (*F* (1, 33) = 70.65, *p* < 0.001, *η*_p_^2^ = 0.68) was observed showing that congruency leads to higher gain/cost score (M = 5.65, SD = 3.96) compared to incongruent encoding (M = −2.138, SD = 4.96). Furthermore, an interaction effect of semantic and modality (*F* (1, 33) = 61.85, *p* < 0.001, *η*_p_^2^ = 0.65) was significant. Subsequent Bonferroni-adjusted *post-hoc* pairwise *t*-tests demonstrated that in the auditory task, encoding of congruent pairings (M = 12.55, SD = 6.99) resulted in significantly higher gain/cost (*t* (34) = 8.87, *p* < 0.001) compared to encoding of incongruent pairings (M = −4.39, SD = 9.32). Conversely, in the visual task, no significant difference was found (*p* > 0.05). Additionally, participants exhibited greater accuracy improvement following congruent encoding (*t* (34) = 7.697, *p* < 0.001) in the auditory task (M = 12.55, SD = 6.99) compared to the visual task (M = −1.24, SD = 5.53). Similarly, incongruence led to a larger decrease in accuracy (*t* (34) = −2.346, *p* = 0.025) in the auditory task (M = −4.393, SD = 9.32) compared to the visual task (M = 0.117, SD = 0.68) (Figure 2A). However, no significant main effect of group was observed (*F* (1, 33) = 0.164, *p* = 0.69), and there was also no interaction with group (Semantic × Group: *F* (1, 33) = 0.098, *p* = 0.75; Modality × Group: *F* (1, 33) = 0.860, *p* = 0.36; Modality × Semantic × Group: *F* (1, 33) = 2.704, *p* = 0.11).

The initial analysis conducted to test the assumptions for the analysis of variance of the gain/cost indices for RT indicated that all conditions exhibited normal distribution, as confirmed by the Shapiro–Wilk test (all *p* > 0.05). The assumption of homogeneity of covariance was satisfied, as determined by Box’s test of covariance matrix equality (*p* = 0.48), and homogeneity of variances was upheld across all conditions, as determined by Levene’s test of error variance equality (all *p* > 0.05). The gain/cost scores of RT were analyzed in the same manner as the analysis of ACC, revealing a significant main effect of modality (*F* (1, 33) = 7.204, *p* = 0.011, *η*_p_^2^ = 0.18), showing a higher gain/cost score in the auditory condition (M = −32.01, SD = 65.49) compared to the visual condition (M = −0.859, SD = 29.52). Furthermore, a significant interaction effects of modality and semantic (*F* (1, 33) = 4.515, *p* = 0.041, *η*_p_^2^ = 0.12) was observed, indicating that in the case of congruent encoding, participants became faster (*t* (34) = 3.717, *p* < 0.001) in the auditory task (M = −46.84, SD = 69.29) compared to the visual task (M = 1.05, SD = 33.50). However, no significant differences were found between the modalities following the encoding of incongruent pairings (*p* > 0.05). Moreover, the interaction of group and semantic (*F* (1, 33) = 4.978, *p* = 0.033, *η*_p_^2^ = 0.13) as well as the interaction of semantic, modality and group were also significant (*F* (1, 33) = 4.78, *p* = 0.020, *η*_p_^2^ = 0.15). Post-hoc t-tests revealed that HC showed a significant increase in RT (*t* (18) = 3.89, *p* < 0.001) in the auditory object recognition task after encoding of congruent pairings (M_A + c_ = −56.59, SD = 76.58) compared to incongruent encoding (M_A + i_ = 11.94, SD = 94.6). However, this effect was not observed in the schizophrenia group (*p* > 0.05, M_A + c_ = −37.09, SD = 62.64; M_A + I_ = −46.31, SD = 75.16) (Figure 2B). To ensure that gain/cost indices significantly differ from zero, each of them was tested against a zero matrix by use of one-tailed independent t-test. This analysis revealed that in the patients’ group the accuracy of A + c (*t* (15) = 9.37, *p* < 0.001), V + c (*t* (15) = −2.38, *p* = 0.027) as well as the gain/cost of reaction time in A + c (*t* (15) = −2.37, *p* = 0.032), A + i (*t* (15) = −2.46, *p* = 0.026) differed from zero. In the HC the accuracy of A + c (*t* (19) = 6.17, *p* < 0.001), A + i (*t* (19) = −2.39, *p* = 0.028) and reaction time of A + c (*t* (19) = −3.30, *p* = 0.004) differed significantly from zero (Figures 2A,B). Furthermore, the values of gain/cost indices are presented in Table 4.

**Table 4:** Gain/cost indices of accuracy and RT for SZ and HC.

	SZ	HC
GCi of ACC %	M (SD)	M (SD)
A + c	14.25 (6.08)	10.84 (7.81)
A + i	−4.31 (11.27)	−4.47 (7.55)
V + c	−2.75 (5.35)	0.26 (5.8)
V + i	0.81 (4.11)	−0.58 (5.61)
**GCi of RT ms**		
A + c	−37.1 (62.64)	−56.59 (76.58)
A + i	−46.31 (75.16)	11.93 (94.6)
V + c	3.90 (40.14)	−1.42 (26.51)
V + i	−0.28 (44.41)	−5.64 (34.5)

To test the linear relationship between patients’ gain/cost indices and their symptoms (measured with PANSS) multiple Pearson Correlations were computed. The result revealed that in the SZ group the gain/cost indices of RT in the incongruent auditory condition negatively correlated with the PANSS total score (*r* (14) = −0.534, *p* = 0.033). However, it’s noteworthy that following the application of the Bonferroni correction to address the concern of multiple comparisons, the correlation no longer retained statistical significance. The correlation between patients’ performance and chlorpromazine equivalence was not significant (*p* > 0.05).

## 4. Discussion

To investigate the interrelation between abnormality in multisensory perception and short term memory impairment in schizophrenia spectrum disorders, we conducted two adapted versions of a continuous recognition task (61, 62). In this paradigm, subjects were presented with initial unimodal and audiovisual pairings that were congruent or incongruent. All task relevant stimuli were presented in a unisensory manner for a second time during the tasks. Participants were asked to indicate whether the stimuli were presented for the first or second time.

The analysis of both ACC and RT showed a significant main effect of modality, indicating a more pronounced gain/cost in the auditory task. This finding is consistent with prior research (19), which suggests that the presentation of task-irrelevant visual stimuli during the encoding of auditory stimuli has a greater impact on subsequent auditory object recognition, as opposed to the influence of task-irrelevant auditory stimuli on visual object recognition. This result supports the principle of “Inverse effectiveness” (73, 74), which proposes that a sensory modality that is less effective at eliciting behaviour for a given task is more likely to exhibit greater multisensory benefits. Since objects are primarily perceived visually, the visual domain provides richer and more reliable object information compared to auditory stimulation (75, 76). This difference explains the observed multimodal enhancement in the auditory task. The interaction effect of modality and semantic revealed that the impact of the semantic relationship between audiovisual pairings during encoding was only evident in the auditory task. Specifically, congruent pairings resulted in a multisensory benefit, while incongruent pairings led to reduced accuracy among participants. In terms of RT, congruent pairing elicited faster response times in both groups. Incongruent pairings did not affect the RT of the HC, while the SZ exhibited even faster RT after encoding the incongruent pairings.

### 4.1. Aberrant object recognition in schizophrenia

Previous studies with healthy subjects using continuous recognition tasks demonstrated that past multisensory experiences, even a short single-trial can influence the subsequent object discrimination. Specifically, recognition is enhanced for congruent multisensory pairings and can be impaired for incongruent pairings (19, 61, 62). The findings of our study regarding the performance of the HC align with these previous observations (19, 61). Furthermore, SZs exhibited a comparable multisensory facilitation effect in the congruent conditions when compared to HC. This finding leads us to reject our hypothesis concerning a diminished multisensory gain in SZ when encoding congruent audiovisual objects within this specific task. However, SZ patients showed also a multisensory facilitation effect, manifested as faster response time, during auditory object recognition in the incongruent condition compared to HC. This outcome aligns with our hypothesis regarding the reduced negative impact of incongruent multisensory pairing and indicates that the multimodal facilitation observed in SZ, in contrast to HC, is independent of the semantic content, highlighting a distinct pattern of processing in this population.

An influential framework that has recently emerged as a promising approach for elucidating the fundamental symptoms of schizophrenia is the predictive coding model (77–81). According to this framework, our brain employs prior beliefs about the environment to make inference about probable causes of sensory inputs. In this view, the brain’s goal is to optimize its internal model about the world by minimizing the discrepancy between expectations encoded in higher processing levels (e.g., top-down signal) and sensory inputs (bottom-up signal) by adjusting the synaptic strength. In each processing step, the sensory signal is compared to the predicted signal and the difference is encoded as a prediction error, which is then used to update prior beliefs, if necessary. The need for model updating (i.e., the tolerated magnitude of the prediction error) varies based on the precision of prior beliefs and/or sensory inputs, thereby balancing the relation of top-down (model predictions) and bottom-up (sensory signals) signals (82). An imbalance on the prediction side (i.e., a strong top-down signal) could even possibly lead to a biased sensory perception (83). Conversely, a strong bottom-up signal would indicate that priors are incorrect and need to be updated (83).

Recent investigations suggest an aberrant balance between the precision of predictions and sensory inputs in SZ (77). SZ has been associated with reduced precision of prior beliefs and/or increased precision of sensory data (84–87). This precision imbalance shifts the perception towards sensory inputs and away from prior beliefs (82). When sensory evidence is given excessive weight, it can result in aberrant saliency of sensory input (84). It is noteworthy that some evidence suggests an opposing perspective, indicating that the concept of loss of prior precision and gain in sensory precision in SZ may not fully account for psychoses (88, 89), as some hallmark symptoms of schizophrenia are associated with strong precision of prior beliefs (90). A recent study (91) investigating auditory perception under different levels of uncertainty showed that the hallucinations in patients with SZ correlated with perceptual bias reflecting increased weighting of prior beliefs. Other studies have demonstrated that patients who experience auditory hallucinations weigh predictions more heavily than sensory evidence as compared to healthy subjects (88, 91, 92).

In what follows, we argue that our results in this specific task provide further support for the notion of reduced precision of prior belief and, therefore, indicate a perceptual shift towards sensory inputs in this patient population.

In addition to the widely recognized factors of spatial and temporal contiguity, the content of stimuli plays a significant role in audio-visual integration (93). Specifically, the semantic attributes of stimuli help determine whether the information conveyed to different senses originates from the same object (20, 76, 93, 94). Lehman and Murray (20) argue that the presentation of semantically congruent auditory–visual objects can lead to formation of distinct perceptual and memory traces. These traces can be quickly reactivated when either the visual or the auditory component is presented again. This suggests that the strength of the prediction/memory determinates the speed at which it resonates with the incoming input and subsequently facilitates recognition. This may occur due to the enhanced activation of a single object representation through multiple sources during the processing of repeated presentation of stimuli (20). EEG and fMRI studies have supported this idea, demonstrating that responses to repeated presentations of unisensory visual or auditory stimuli are influenced at early latencies (60 ms after stimulus onset) by whether these stimuli were previously presented in conjunction with a sound or image (19). In contrast, semantically incongruent pairings can be simultaneously encoded via distributed neuronal representations as separate objects (20, 95) leading to the absence of multisensory enhancement. However, our results demonstrated a RT facilitation effect in the incongruent condition for the SZ, which is comparable to the RT facilitation observed in the HC and SZ for the congruent condition. Our findings lend support to the proposition that the imbalance in prediction error and reliance on weak prior beliefs and/or robust bottom-up signals in SZ contribute to an increased reliance on the mere spatial and temporal concurrence of sensory inputs for the formation of perceptual object units. This may lead to temporary integration of incongruent auditory and visual stimuli and therefore, encoding them as a unitary object through multisensory traces. This may be beneficial in some cases, as patients showed faster RT in the incongruent condition in the current study. However, it could also overwhelm patients with information flows, considering that our environment is full of simultaneous stimuli that are semantically unrelated to each other. Given our study’s behavioral focus, further neuroimaging investigations are necessary to validate this proposition.

An essential feature of predictive coding is its hierarchical structure (80). Consequently, the precision weighting of prediction errors occurs independently at different hierarchical levels and across various sensory modalities (80). As a result, drawing a definitive conclusion about the specific direction of precision weighting for prediction errors through behavioral data becomes challenging, highlighting the pivotal role of incorporating neuroimaging techniques. Furthermore, the utilization of neuroimaging methodologies has the potential to provide a deeper understanding of the diverse impacts of precision imbalances on the processes of encoding and recall. This opens up a captivating and promising avenue for future investigations.

Although, our study included a unisensory condition in both tasks, incorporating a multisensory meaningless condition (e.g., geometric figures and noise), could provide further evidence about the strength of prior beliefs and sensory signal in SZ. While the generalizability of our findings may be constrained by the small sample size of participants, our *post hoc* power analysis, performed using G*Power 3.1.9.7 (96), revealed that all main and interaction effects surpassed the 80 percent threshold in statistical power (see Supplementary Table S1 for more details). To bolster the robustness of our results, future research endeavors should prioritize replication with a larger sample size Lastly, considering the heterogeneity of symptoms in SZs and prior research indicating a connection between distinct symptoms like auditory hallucinations and pronounced top-down effect (88, 91, 92), exploring audiovisual integration across various subtypes of schizophrenia could offer valuable insights for future studies.

## 5. Conclusion

In accordance with previous studies (19, 61, 62), we demonstrated that the effect of task-irrelevant stimuli during encoding is still observable at least after 9 ± 4 trials and can be differentiated based on the semantical relationship between the stimuli presented during encoding. This effect was more pronounced in the auditory task, where participants focused on the auditory stimulus. While SZs exhibited a similar performance profile to HC under multisensory congruent conditions, implying intact congruent audiovisual integration within the utilized continuous object recognition task, a notable distinction was observed. Unlike HC, individuals with SZ exhibited a multisensory facilitation effect, manifested as faster RT, after being initially encountered with incongruent pairings in the auditory task. Based on the predictive coding model, we suggest that this observed deviation indicate a reduced semantic modulatory effect and anomalous prediction error signalling, particularly in the context of semantically conflicting cross-modal sensory inputs in SZ.

## Data availability statement

The raw data supporting the conclusions of this article will be made available by the authors, without undue reservation.

## Ethics statement

The studies involving humans were approved by the Ethics Committee of Hannover Medical School. The studies were conducted in accordance with the local legislation and institutional requirements. Written informed consent for participation in this study was provided by the participants’ legal guardians/next of kin.

## Author contributions

EG, CS, and GS conceived and coordinated the study. EG, AB, and CS performed the statistical analysis and interpretation of the data. EG performed the measurement and the diagnostic investigation. EG and AB drafted the manuscript. CS, GS, and SB reviewed the manuscript. AČ participated in the coordination of study, supported the statistical analysis and the data interpretation. All authors contributed to the article and approved the submitted version.
