# Supplementary material for: The effect of multisensory semantic congruency on unisensory object recognition in schizophrenia
Source: Front Psychiatry. 2023 Nov 1;14:1246879. doi: 10.3389/fpsyt.2023.1246879 (PMC10646423; doi:10.3389/fpsyt.2023.1246879)
Supplement: Supplementary file 1 [file Table_1.DOCX]

Supplementary Material

The Effect of Multisensory Semantic Congruency on Unisensory Object Recognition in Schizophrenia

**Erfan Ghaneirad*, Anna Borgolte, Christopher Sinke, Anja Čuš, Stefan Bleich, Gregor R. Szycik**

*** Correspondence: Ghaneirad.seyederfan@mh-hannover.de**

| **Gain/cost indices of ACC** | | | **Gain/Cost indices of RT** | | |
| --- | --- | --- | --- | --- | --- |
|  | Effect size f |  |  | Effect size f |  |
| Modality | 0.57 | 99% | Modality | 0.46 | 99% |
| Semantic | 1.45 | 100% | Modality*semantic | 0.36 | 98% |
| Semantic*Modality | 1.36 | 100% | Group*Semantic | 0.38 | 99% |
|  |  |  | Semantic*Modality*Group | 0.42 | 99% |

**Supplementary Table 1: Post Hoc Power Analysis Details.** This table presents the results of a post hoc power analysis conducted using G*Power 3.1.9.7. The analysis utilized a repeated measures ANOVA with a within-between interaction design and was carried out at a significance level (alpha) of 0.05. The degrees of freedom are as follows: numerator df = 1 and denominator df = 33.
